# Supplementary material for: Unveiling the Bioactive Potential of the Invasive Jellyfish Phyllorhiza punctata Through Integrative Transcriptomic and Proteomic Analyses
Source: Biomolecules. 2025 Aug 4;15(8):1121. doi: 10.3390/biom15081121 (PMC12383608; doi:10.3390/biom15081121)
Supplement: Supplementary file 1 [file biomolecules-15-01121-s001.zip › Suplementary Material/FileS3/PEPFOLD4/XP_065058404_12221/clusters.html]

 PEP-FOLD clusters


Download archive of all models  here   

|  |  |  |
| --- | --- | --- |
| # | file | sOPEP |
| # cluster 1 - Size: 2 |  |  |
|  | XP\_065058404\_12221-model1 | -17.3899 |
|  | XP\_065058404\_12221-model1.1 | -9.95649 |
| # cluster 2 - Size: 2 |  |  |
|  | XP\_065058404\_12221-model2 | -16.9458 |
|  | XP\_065058404\_12221-model2.1 | -11.3837 |
| # cluster 3 - Size: 1 |  |  |
|  | XP\_065058404\_12221-model3 | -15.4625 |
| # cluster 4 - Size: 1 |  |  |
|  | XP\_065058404\_12221-model4 | -15.375 |
| # cluster 5 - Size: 9 |  |  |
|  | XP\_065058404\_12221-model5 | -15.1567 |
|  | XP\_065058404\_12221-model5.1 | -11.9468 |
|  | XP\_065058404\_12221-model5.2 | -11.15 |
|  | XP\_065058404\_12221-model5.3 | -11.0898 |
|  | XP\_065058404\_12221-model5.4 | -11.0523 |
|  | XP\_065058404\_12221-model5.5 | -9.3663 |
|  | XP\_065058404\_12221-model5.6 | -9.18412 |
|  | XP\_065058404\_12221-model5.7 | -9.05776 |
|  | XP\_065058404\_12221-model5.8 | -8.86953 |
| # cluster 6 - Size: 3 |  |  |
|  | XP\_065058404\_12221-model6 | -14.5436 |
|  | XP\_065058404\_12221-model6.1 | -12.0723 |
|  | XP\_065058404\_12221-model6.2 | -11.9569 |
| # cluster 7 - Size: 1 |  |  |
|  | XP\_065058404\_12221-model7 | -14.4765 |
| # cluster 8 - Size: 9 |  |  |
|  | XP\_065058404\_12221-model8 | -14.2311 |
|  | XP\_065058404\_12221-model8.1 | -11.7328 |
|  | XP\_065058404\_12221-model8.2 | -11.6061 |
|  | XP\_065058404\_12221-model8.3 | -10.5347 |
|  | XP\_065058404\_12221-model8.4 | -9.93939 |
|  | XP\_065058404\_12221-model8.5 | -9.54905 |
|  | XP\_065058404\_12221-model8.6 | -9.42149 |
|  | XP\_065058404\_12221-model8.7 | -8.95758 |
|  | XP\_065058404\_12221-model8.8 | -8.35527 |
| # cluster 9 - Size: 4 |  |  |
|  | XP\_065058404\_12221-model9 | -13.803 |
|  | XP\_065058404\_12221-model9.1 | -13.7528 |
|  | XP\_065058404\_12221-model9.2 | -13.0103 |
|  | XP\_065058404\_12221-model9.3 | -11.3075 |
| # cluster 10 - Size: 1 |  |  |
|  | XP\_065058404\_12221-model10 | -13.7777 |
| # cluster 11 - Size: 1 |  |  |
|  | XP\_065058404\_12221-model11 | -13.7305 |
| # cluster 12 - Size: 1 |  |  |
|  | XP\_065058404\_12221-model12 | -13.5775 |
| # cluster 13 - Size: 2 |  |  |
|  | XP\_065058404\_12221-model13 | -13.552 |
|  | XP\_065058404\_12221-model13.1 | -11.0403 |
| # cluster 14 - Size: 2 |  |  |
|  | XP\_065058404\_12221-model14 | -13.5462 |
|  | XP\_065058404\_12221-model14.1 | -8.08639 |
| # cluster 15 - Size: 1 |  |  |
|  | XP\_065058404\_12221-model15 | -13.3858 |
| # cluster 16 - Size: 3 |  |  |
|  | XP\_065058404\_12221-model16 | -13.3506 |
|  | XP\_065058404\_12221-model16.1 | -10.2621 |
|  | XP\_065058404\_12221-model16.2 | -9.57146 |
| # cluster 17 - Size: 1 |  |  |
|  | XP\_065058404\_12221-model17 | -12.637 |
| # cluster 18 - Size: 1 |  |  |
|  | XP\_065058404\_12221-model18 | -12.0771 |
| # cluster 19 - Size: 3 |  |  |
|  | XP\_065058404\_12221-model19 | -11.7996 |
|  | XP\_065058404\_12221-model19.1 | -10.597 |
|  | XP\_065058404\_12221-model19.2 | -10.0502 |
| # cluster 20 - Size: 5 |  |  |
|  | XP\_065058404\_12221-model20 | -11.7755 |
|  | XP\_065058404\_12221-model20.1 | -9.3471 |
|  | XP\_065058404\_12221-model20.2 | -9.17718 |
|  | XP\_065058404\_12221-model20.3 | -8.4858 |
|  | XP\_065058404\_12221-model20.4 | -7.90275 |
| # cluster 21 - Size: 2 |  |  |
|  | XP\_065058404\_12221-model21 | -11.7735 |
|  | XP\_065058404\_12221-model21.1 | -11.0707 |
| # cluster 22 - Size: 4 |  |  |
|  | XP\_065058404\_12221-model22 | -11.5829 |
|  | XP\_065058404\_12221-model22.1 | -9.90796 |
|  | XP\_065058404\_12221-model22.2 | -8.12138 |
|  | XP\_065058404\_12221-model22.3 | -8.00263 |
| # cluster 23 - Size: 1 |  |  |
|  | XP\_065058404\_12221-model23 | -11.559 |
| # cluster 24 - Size: 2 |  |  |
|  | XP\_065058404\_12221-model24 | -11.542 |
|  | XP\_065058404\_12221-model24.1 | -8.08361 |
| # cluster 25 - Size: 1 |  |  |
|  | XP\_065058404\_12221-model25 | -11.4783 |
| # cluster 26 - Size: 1 |  |  |
|  | XP\_065058404\_12221-model26 | -11.409 |
| # cluster 27 - Size: 7 |  |  |
|  | XP\_065058404\_12221-model27 | -11.3907 |
|  | XP\_065058404\_12221-model27.1 | -10.8466 |
|  | XP\_065058404\_12221-model27.2 | -10.3552 |
|  | XP\_065058404\_12221-model27.3 | -10.2566 |
|  | XP\_065058404\_12221-model27.4 | -9.9826 |
|  | XP\_065058404\_12221-model27.5 | -9.27124 |
|  | XP\_065058404\_12221-model27.6 | -8.36022 |
| # cluster 28 - Size: 1 |  |  |
|  | XP\_065058404\_12221-model28 | -10.8028 |
| # cluster 29 - Size: 1 |  |  |
|  | XP\_065058404\_12221-model29 | -10.4544 |
| # cluster 30 - Size: 2 |  |  |
|  | XP\_065058404\_12221-model30 | -10.3361 |
|  | XP\_065058404\_12221-model30.1 | -8.63781 |
| # cluster 31 - Size: 2 |  |  |
|  | XP\_065058404\_12221-model31 | -10.2615 |
|  | XP\_065058404\_12221-model31.1 | -8.0364 |
| # cluster 32 - Size: 3 |  |  |
|  | XP\_065058404\_12221-model32 | -10.1631 |
|  | XP\_065058404\_12221-model32.1 | -9.85465 |
|  | XP\_065058404\_12221-model32.2 | -8.73524 |
| # cluster 33 - Size: 1 |  |  |
|  | XP\_065058404\_12221-model33 | -10.026 |
| # cluster 34 - Size: 2 |  |  |
|  | XP\_065058404\_12221-model34 | -9.92804 |
|  | XP\_065058404\_12221-model34.1 | -9.23033 |
| # cluster 35 - Size: 1 |  |  |
|  | XP\_065058404\_12221-model35 | -9.87035 |
| # cluster 36 - Size: 2 |  |  |
|  | XP\_065058404\_12221-model36 | -9.84051 |
|  | XP\_065058404\_12221-model36.1 | -7.95194 |
| # cluster 37 - Size: 1 |  |  |
|  | XP\_065058404\_12221-model37 | -9.58982 |
| # cluster 38 - Size: 1 |  |  |
|  | XP\_065058404\_12221-model38 | -9.4575 |
| # cluster 39 - Size: 1 |  |  |
|  | XP\_065058404\_12221-model39 | -9.4161 |
| # cluster 40 - Size: 1 |  |  |
|  | XP\_065058404\_12221-model40 | -9.35603 |
| # cluster 41 - Size: 1 |  |  |
|  | XP\_065058404\_12221-model41 | -9.34555 |
| # cluster 42 - Size: 1 |  |  |
|  | XP\_065058404\_12221-model42 | -9.01982 |
| # cluster 43 - Size: 2 |  |  |
|  | XP\_065058404\_12221-model43 | -8.41157 |
|  | XP\_065058404\_12221-model43.1 | -8.17189 |
| # cluster 44 - Size: 1 |  |  |
|  | XP\_065058404\_12221-model44 | -8.37359 |
| # cluster 45 - Size: 1 |  |  |
|  | XP\_065058404\_12221-model45 | -8.19368 |
| # cluster 46 - Size: 1 |  |  |
|  | XP\_065058404\_12221-model46 | -8.17617 |
| # cluster 47 - Size: 1 |  |  |
|  | XP\_065058404\_12221-model47 | -8.09929 |
| # cluster 48 - Size: 1 |  |  |
|  | XP\_065058404\_12221-model48 | -7.9804 |
| # cluster 49 - Size: 1 |  |  |
|  | XP\_065058404\_12221-model49 | -7.89495 |
|  |  |  |
